# Forest Restoration in Low- and Middle-Income Countries

**DOI:** 10.1146/annurev-environ-012220-020159

**Published:** 2021-05-19

**Authors:** Jeffrey R. Vincent, Sara R. Curran, Mark S. Ashton

**Affiliations:** 1 Nicholas School of the Environment, Duke University, Durham, North Carolina 27708, USA; 2 Henry M. Jackson School of International Studies, University of Washington, Seattle, Washington 98195, USA; 3 The Forest School at the Yale School of the Environment, Yale University, New Haven, Connecticut 06511, USA

**Keywords:** forest restoration, forest transition, afforestation, reforestation, migration, payments for ecosystem services

## Abstract

A series of international initiatives have set ambitious goals for restoring global forests. This review synthesizes natural and social science research on forest restoration (FR), with a focus on restoration on cleared land in low- and middle-income countries. We define restoration more broadly than reestablishing native forests, given that landholders might prefer other forest types. We organize the review loosely around ideas in the forest transition literature. We begin by examining recent trends in FR and forest transition indicators. We then investigate two primary parts of the forest transition explanation for forest recovery: wood scarcity, including its connection to restoration for climate change mitigation, and the dynamic relationships between migration and land use. Next, we review ecological and silvicultural aspects of restoration on cleared land. We conclude by discussing selected interventions to promote restoration and the challenge of scaling up restoration to achieve international initiatives’ goals.

## 1. INTRODUCTION

Since 2010, a series of international initiatives have set ambitious goals for restoring the world’s forests. The Bonn Challenge, launched in 2011, aims at restoring 350 million hectares (Mha) by 2030 (http://www.bonnchallenge.org/). In 2019, the United Nations (UN) General Assembly announced the UN Decade on Ecosystem Restoration 2021–2030 to accelerate progress toward the Bonn Challenge (http://www.decadeonrestoration.org/). In 2020, the World Economic Forum created a common platform to unite various forest restoration (FR) initiatives and support the UN Decade (https://www.1t.org/). Climate change mitigation, biodiversity conservation, and enhanced rural livelihoods feature as prominent objectives of these initiatives.

Across a landscape, FR might entail a range of actions on a variety of lands owned or used by diverse landholders, from households and communities to businesses and governments (1). It might occur as a planned process that coordinates actions across landholders, or it might occur spontaneously through landholders’ decentralized decisions. It might include rehabilitating existing but degraded forests to full productivity, or reestablishing forests on land cleared decades or centuries earlier for other uses. It might recreate forests with characteristics matching those of the native forests originally found on the sites, i.e., restoration in a literal sense. Or, it might deliberately create forests with different characteristics, such as plantations of introduced (non-native) species, to prioritize specific goods and services that landholders value.

This article reviews FR on cleared land in regions where forests would naturally occur, with forest (2) defined in a general way that encompasses restoration in both the literal and broader senses just discussed. Given the focus on cleared land, the term FR as used here refers more to afforestation than reforestation, although some topics covered are relevant to reforestation after unintended events, such as wildfires, that cause a loss of forest cover but not a change in land use. The article is primarily concerned with FR on land cleared for agriculture, not land uses that cause more severe degradation (e.g., mining). Land clearing for agriculture has been the primary cause of forest cover loss throughout history (3), and agricultural land (cropland plus pastureland) thus comprises the largest stock of potential land for FR. Indeed, the largest government-sponsored FR program in the world, China’s Sloping Lands Conversion Program (SLCP), targets agricultural land (4), as does Brazil’s similarly ambitious National Plan for Native Vegetation Recovery (5).

Our review adopts a geographical focus: low- and middle-income countries (LMICs) in Africa, Asia and the Pacific, and Latin America and the Caribbean. These countries are located mainly in the tropics and subtropics. We organize our review of FR in LMICs loosely around ideas in the forest transition literature (6, 7). This literature was motivated by the observation that much agricultural land in today’s high-income countries reverted to forest during the previous century. It offers two interrelated processes as the primary explanations for forest recovery: (*a*) industrialization, which draws migrant labor out of rural areas into cities and shifts rural land toward less labor-intensive uses, including forestry, and (*b*) wood scarcity, which creates an incentive for landholders to plant or otherwise regenerate trees, especially on less productive land. It emphasizes that the transition is not an automatic feature of economic development and is mediated by various factors, including property rights, international trade, forestry policies, and public demand for environmental protection. We focus on LMICs because they are emphasized by international FR initiatives and are where the forest transition is most uncertain (8).

We begin by examining recent trends in FR and variables associated with the forest transition in LMICs, and we comment on the trends’ implications for achieving 2030 FR targets (Section 2). We then examine information on the two primary parts of the forest transition explanation for forest recovery: wood scarcity, including its connection to FR as a climate mitigation strategy (Section 3), and the dynamic relationships between migration and land use (Section 4). Next, we review methods for restoring forests on cleared land (Section 5). We conclude by discussing selected interventions to promote FR (Section 6) and the challenge of scaling up FR (Section 7).

Our review of socioeconomic aspects of FR relies when possible on studies that apply impact evaluation methods, which aim at quantifying an intervention’s causal effect on specified outcomes. Although these studies attempt to control for methodological sources of bias that can undermine causal inference, previous reviews caution that biases might remain in many impact evaluation studies relevant to FR (9–11). We also note that the economic literature on FR is relatively thin, and thinner than the biophysical literature, because economic research on forest-cover change in LMICs has focused more on deforestation than on FR (12, 13). Even benefit-cost analyses of FR projects are few and flawed (14). For these reasons, we occasionally draw on socioeconomic studies from the broader literature on agroforestry, although systematic coverage of that topic is beyond the scope of this review.

## 2. FOREST RESTORATION AND FOREST TRANSITION TRENDS

National statistics in the 2020 *Global Forest Resources Assessment* (15) (FRA) by the UN Food and Agriculture Organization (FAO) paint a broad-brush picture of recent FR trends in the 139 LMICs in Africa, Asia and the Pacific, and Latin America and the Caribbean that we focus on. FRA provides data on total forest area in 2000, 2010, and 2020 for all 139 countries and data on planted forest area for 127 of them. We performed a simple data quality check before analyzing trends in the data: We excluded data points if the reported areas did not change between time points, as FAO’s practice is to carry forward earlier estimates when it lacks updated ones. This reduced the sample to 112 countries for total forest area and 79 countries for planted forest area.

Total forest area increased during 2000–2020 in 32 of the 112 countries, whereas planted forest area increased in 67 of the 79 countries. Data on planted area are available for 26 of the 32 countries where total area increased. For this subset of countries, the aggregate increase in planted area was equivalent to 68% of the aggregate increase in total area. This percentage suggests that active restoration was responsible for more of the increase in total area than passive restoration was.

**Figure 1:**
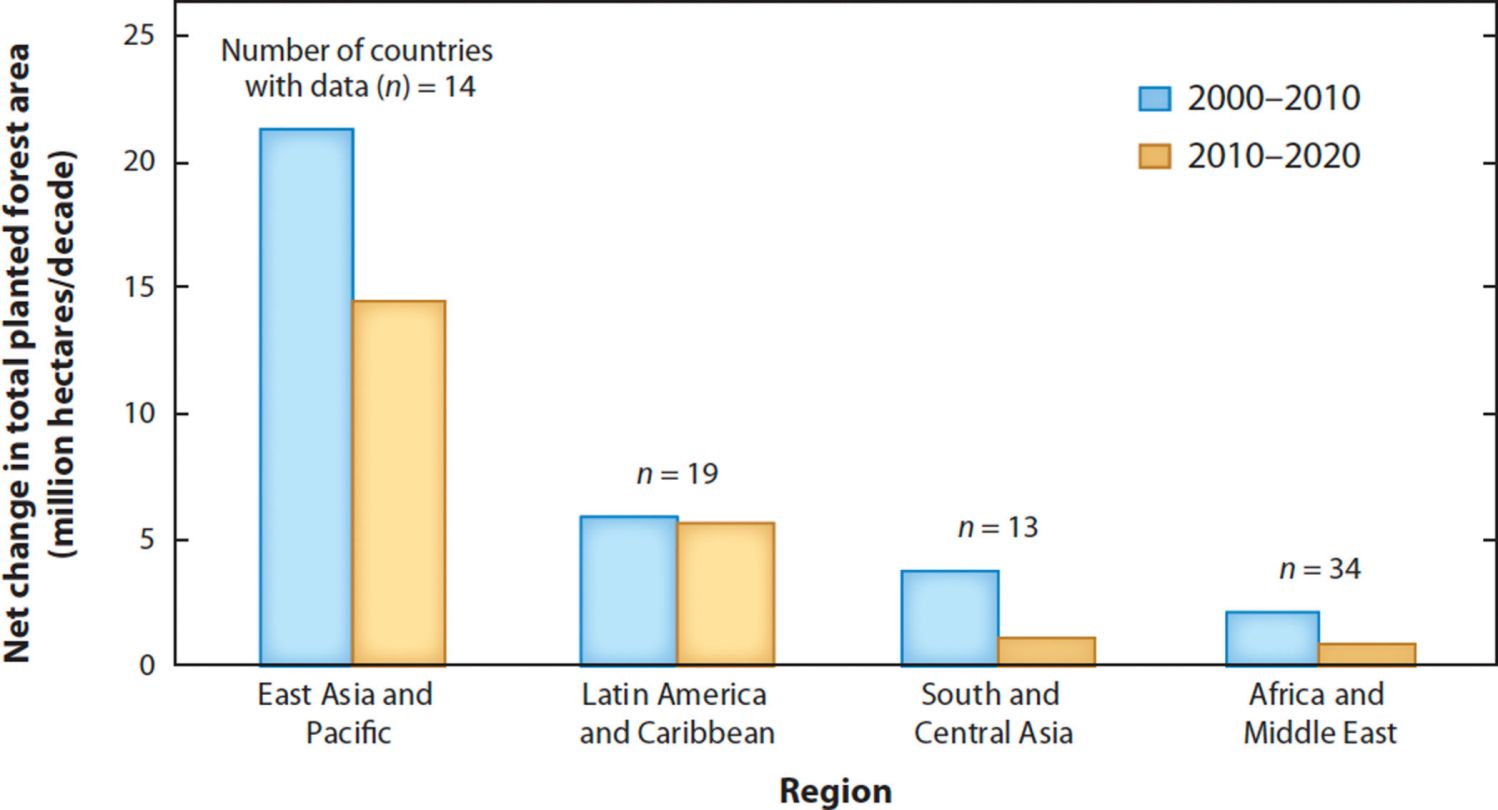
Net change in total planted forest area in low- and middle-income countries by region and decade, calculated as total area at end of decade minus total area at beginning of decade. Total planted forest area increased in all regions in both decades, but it increased more slowly during the second decade, especially outside of Latin America and the Caribbean. Regional totals include only countries with data on total planted forest area in all three years, 2000, 2010, and 2020. Data from Reference 15.

These trends suggest that forest transitions are underway in many LMICs. Examining data separately for 2000–2010 and 2010–2020 reveals mixed evidence on the pace of transition, however. Although the number of countries with increasing total forest area rose slightly from 29 during 2000–2010 to 32 during 2010–2020, planted forest area increased more slowly during the second decade in all LMIC regions (Figure 1). This deceleration is not encouraging evidence of the impact of international FR initiatives launched since 2010.

Furthermore, comparing national trends in the FRA data during 2010–2020 to FR pledges that national governments have made for 2015–2030 under the Bonn Challenge and other international restoration programs (16) indicates that realizing the pledges will require a massive scaling up of FR. For 51 LMICs with data on both FR pledges and total forest area, the average national pledge prorated to 10 years, 3.4 Mha, compares to an average loss of 0.7 Mha of total forest area during 2010–2020. For 41 LMICs with data on both FR pledges and planted forest area, the average national pledge prorated to 10 years, 3.5 Mha, is seven times the average increase in planted forest area during 2010–2020, 0.5 Mha.

**Figure 2:**
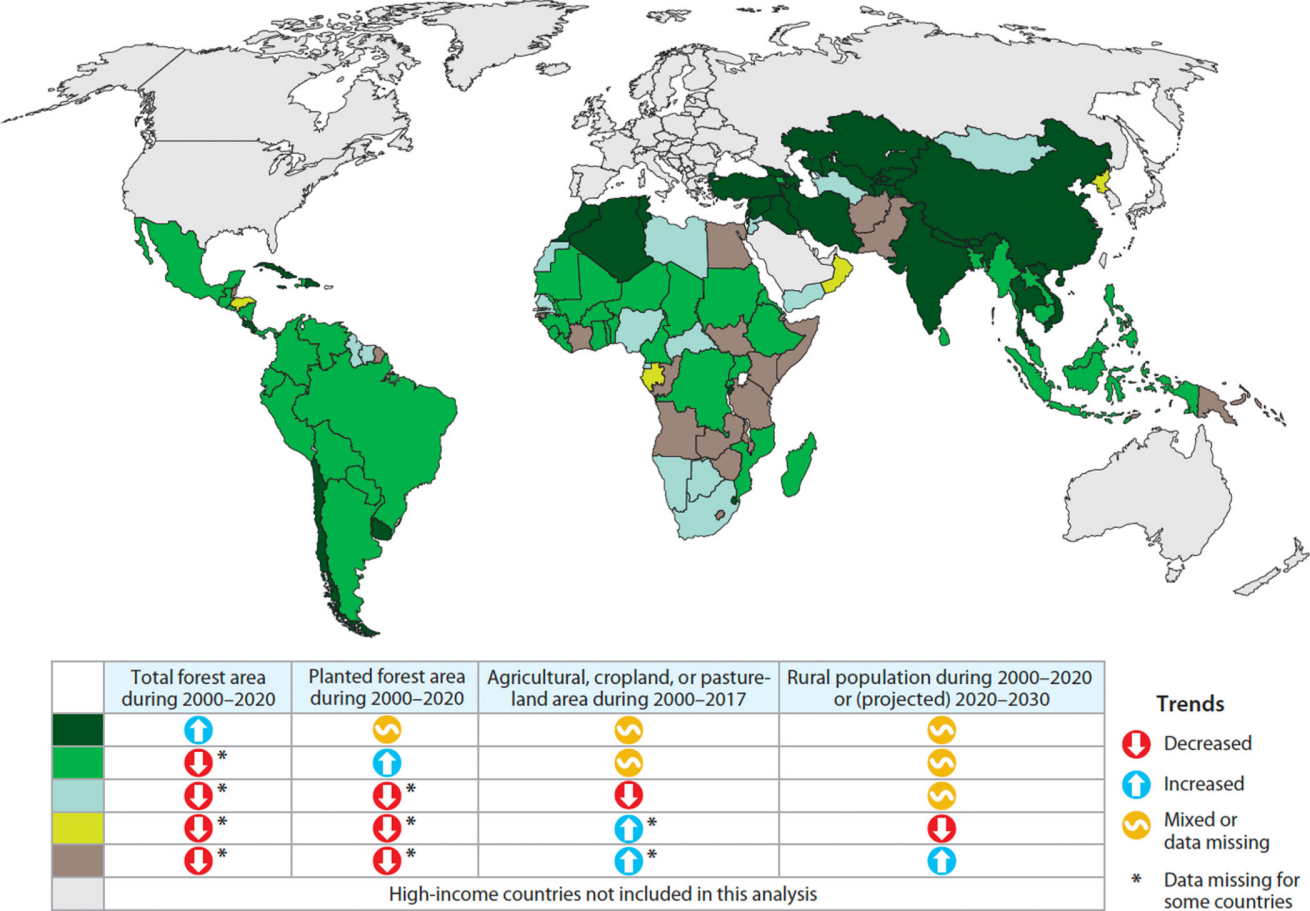
Evidence of a forest transition in low- and middle-income countries (LMICs) in Africa, Asia and the Pacific, and Latin America and the Caribbean. (*Dark green*) Countries with increased total forest area during 2000–2020. (*Green*) Countries with increased planted forest area during 2000–2020, but not increased total forest area. (*Light blue-green*) Countries with decreased agricultural, cropland, or pastureland area during 2000–2017, but not increased total or planted forest area during 2000–2020. (*Light yellow-green*) Countries with decreased rural population during 2000–2020 or (projected) 2020–2030, but not increased total or planted forest area during 2000–2020 or decreased agricultural, cropland, or pastureland area during 2000–2017. (*Brown*) Remaining LMICs. Data from sources described in text.

More encouragingly, national statistics on agricultural areas in 2000 and 2017 (http://www.fao.org/faostat/en/#home; 2020 data are not available) and rural populations in 2000, 2020, and forecast to 2030 (https://population.un.org/wup/) suggest that forest transition conditions are emerging in some LMICs where total or planted forest areas have not yet increased (Figure 2). After performing the data quality check described above, we determined the 2000–2017 change in agricultural area for the 130 LMICs with data. Agricultural area declined in 54 countries, and either cropland or pastureland (the two components of agricultural land) declined in an additional 20 countries. Rural population data are available for all 139 LMICs. Rural populations declined in 35 countries during 2000–2020 and are forecast to decline in an additional 20 countries by 2030. In the next two sections, we first consider whether wood scarcity, on its own or in combination with carbon pricing policies, is likely to induce much future FR on agricultural land in LMICs, and we then examine the complex relationships among rural populations, land degradation, and FR.

## 3. WOOD SCARCITY AND CARBON PRICING

The total global land area where trees can grow but do not already exist, or are unlikely to displace existing land uses (e.g., urban areas), is much larger than the Bonn Challenge’s 0.35 billion hectares (Bha) goal. Estimates of this biophysically restorable area are ∼2 Bha (17, 18), which is equivalent to half of current global forest area (15). LMICs account for a large share of it, approximately half to two-thirds according to our tabulation of country-level estimates reported in the original studies (17, 18). The actual biophysically restorable area might be even larger, as the studies exclude FR on cropland (but not pastureland), out of concern for food security. Adding cropland that has been taken permanently out of production (abandoned land) or has low yields (marginal land) would increase the global area by ∼0.5 Bha (19–21).

Below, we consider whether wood scarcity and carbon pricing can create sufficiently strong incentives for landholders to restore such large areas. We also review evidence on associated food impacts.

### 3.1. Projecting Forest Restoration to 2100

At different times, rising wood scarcity has created an economic incentive for FR in countries as disparate as India, the United States, and nations in West Africa (6). This incentive has weakened globally since 1990. Investments in high-yielding wood plantations and management of natural forests have caused the global wood inventory, as measured by standing wood volume in the world’s forests, to remain nearly stable between 1990 (560 billion m^3^) and 2020 (557 billion m^3^) despite continuing deforestation (15). This stability implies a global balance between wood supply and demand, with the consequence that timber prices have not increased at the rapid rates that economic models of global wood markets predicted in the late 1980s (22). These models compare the value of land for wood production to the land’s opportunity cost—its value in its alternative use, typically agriculture—and allocate land to the highest valued use. The market incentive for landholders to increase forest area is weaker if timber prices rise less rapidly.

A recent study based on one leading model, the Global Timber Model (GTM), predicts that wood scarcity driven by future demand for conventional wood products (lumber, plywood and other panel products, pulp and paper products) will not provide a strong incentive for global FR during the remainder of the century (23). It predicts that global forest area will barely change between 2010 and 2100: a net increase of less than 0.1 Bha. Wood scarcity will evidently enable forests to stand their ground against agriculture but not gain appreciable area.

Harnessing wood markets to scale up FR requires increasing the demand for wood. Concern about climate change has prompted research on expanded use of wood as a substitute for more carbon-intensive materials. Examples include substituting wood for cement and steel in high-rise construction (24), petrochemicals in plastics production (25), and fossil fuels in energy production (26). The GTM study investigated the latter, with expanded global use of woody bioenergy occurring in the context of a climate policy that limited warming to 1.5°C. The hypothetical policy combined a global carbon tax on all emissions sources, including woody bioenergy, with a subsidy for forest carbon sequestration. The study predicted that 2100 global forest area would be 1.6 Bha higher in this scenario than in a business-as-usual (BAU) scenario of conventional wood demand with no carbon policy. This difference approaches the ∼2 Bha estimates of biophysically restorable areas (17, 18).

The GTM study does not provide country-level predictions, as it divides the world into just 16 regions, but it reports aggregate predictions for the tropics. Under BAU, tropical forest area falls by a small amount during 2010–2100 (<0.1 Bha, compared to a total 2010 area of 1.1 Bha), but under the tax cum subsidy policy it is 0.6 Bha higher in 2100 than under BAU. Increased wood demand combined with a policy that rewards forest carbon sequestration evidently could propel substantial FR in tropical LMICs.

### 3.2. Environmental and Social Impacts of Wood Plantations

Large-scale FR driven by wood markets has raised concerns about potentially negative environmental and social impacts (27, 28). Although converting annual cropland to periodically harvested forests might incidentally supply some nontimber forest services (e.g., reduced erosion, improved water quality), FR driven by wood markets can be expected to undersupply many services because markets typically do not compensate landholders for supplying them (27). Increased forest carbon sequestration under the carbon tax cum subsidy policy in the GTM study illustrates the environmental benefits that can result from compensating landholders for supplying nontimber services. So does the study’s predicted composition of the additional 0.6 Bha of 2100 tropical forest area under that policy: Most of the additional area is unharvested natural forests (78%), not harvested natural forests (17%) or wood plantations (5%). Although the policy is linked to a single nontimber forest service, i.e., carbon sequestration, the expansion of unharvested natural forests would supply additional environmental benefits too.

Undersupply of nonmarket forest ecosystem services has been a particular concern for wood plantations, especially if plantations replace natural forests. Both aggregate statistics and studies in individual countries indicate that the risk of the latter occurring is not great. Planted forests, a broader category than plantations, accounted for only 8% of 2020 LMIC forest area (15). The GTM study projects that the share of plantations in total tropical forest area would increase by just 1.5 percentage points between 2010 and 2100 under BAU and even less under the carbon tax cum subsidy policy. In China, which experienced the largest increase in total forest area of any country (not only LMICs) during 2000–2020, remote sensing reveals most of the increase resulted from conversion of cropland to planted forests (29). An impact evaluation of Chile’s Decree Law 701, which provides a package of incentives for wood plantations, found that the law roughly doubled smallholder plantation area during 1998–2012, but mainly through pastureland conversion (30), not conversion of native forests. A simulation study estimated that the law was responsible for just 5% of native forest loss during 1986–2011, with only tiny effects on aboveground carbon storage and biodiversity (31). An impact evaluation in Uganda found that the leasing of public forestland to private investors for commercial wood plantations actually had a positive environmental spillover, as it reduced the extraction of fuelwood and other forest products from natural forests (32).

Only approximately a quarter of studies on the social impacts of wood plantations in LMICs have attempted to control for sources of methodological bias (28). Within that subset of more rigorous studies, a larger number reported that plantations improved local livelihoods than harmed them, although a majority found that plantations negatively affected the social fabric (e.g., they increased conflict). Many of the studies likely underestimated plantations’ long-term economic benefits to local communities, because they analyzed time periods shorter than ones required for communities to receive those benefits (28).

### 3.3. Food Security

Large-scale FR driven by carbon pricing has raised concerns about negative impacts on food security. A recent study used the Global Trade Analysis Project (GTAP) model to investigate the impacts of a carbon tax cum forest carbon subsidy policy on food prices (33). Compared to the GTM, the GTAP model represents the global food sector in more detail and the global forest sector in less detail. The GTAP study analyzed a less stringent climate policy than the GTM study did, with warming limited to 2°C instead of 1.5°C, yet it predicted that the conversion of agricultural land to forest would raise food prices by 3–4 times in most regions. It warned that such large food-price increases could make FR socially and politically unacceptable as a climate mitigation strategy.

Similar to the GTM, however, the GTAP model has low spatial resolution, which might cause it to overestimate food-price increases if FR occurs on less productive agricultural land that is effectively hidden within its aggregate regions. A study on the food impacts of China’s SLCP based on a higher resolution model concluded that the SLCP had negligible impacts on grain production and prices (34). A study in the Americas found that allowing the effect of agricultural returns on land-use transitions to vary across grid cells instead of being identical substantially altered predictions of transitions between agriculture and natural cover, including forests (35).

### 3.4. Spatially Disaggregated Restoration Cost Studies

The low resolution of market models like the GTM and the GTAP model reduces their usefulness to governments and other organizations seeking information on areas within a given country where FR is feasible under carbon pricing and other policies. Three alternative modeling approaches provide higher resolution information, although they sacrifice the economic adjustments embodied in market models. The simplest approach divides a landscape into units, such as grid cells, and estimates the FR cost and other characteristics of each unit (36). Particularly interesting examples of this approach are spatial optimization models (37), which apply mathematical programming to determine cost-effective FR plans: the set of spatial units that minimizes the aggregate cost of achieving specific restoration objectives, such as carbon sequestration or biodiversity conservation. A recent global exercise estimates that spatial optimization can reduce by 27% the aggregate cost of restoring 15% of global agricultural land to forest (38).

The second approach allows for cost variation within spatial units, by assigning cost curves to them. It enables one to estimate the amount of land within each unit that can be feasibly restored at different levels of compensation offered to landholders, with landholders assumed to restore only land where compensation at least matches the cost. A study on natural climate solutions estimated that, compared to BAU (i.e., no price on carbon), a carbon price of US$100/ton CO_2_e would provide sufficient compensation to offset the cost of restoring 0.2 Bha of forest globally by 2030, with 0.14 Bha being in the tropics (39). The 0.2 Bha global estimate is roughly consistent with the Bonn Challenge’s 0.35 Bha goal, given that the latter includes rehabilitation of degraded forests in addition to FR on cleared land.

The final approach recognizes that compensation exceeding restoration cost might not induce landholders to restore their land, as they can have nonpecuniary reasons for retaining land in its current use or might want to retain an option for a future nonforestry use (40). A recent study of this type revisited the previous study’s 0.14 Bha FR estimate for the tropics, using statistical evidence on the observed impact of agricultural returns on the probability of land-use change between forest and agriculture (41). It concluded that a US$100/ton CO_2_e carbon price would induce only 0.05 Bha of tropical FR by 2030 compared to BAU. Carbon prices evidently must be higher than US$100/ton CO_2_e to incentivize large-scale FR in the tropics.

## 4. DEMOGRAPHIC ASPECTS OF FOREST RESTORATION

The FR studies just reviewed scarcely mention migration, despite its central role in the forest transition literature. Land degradation and FR both influence and are influenced by human population dynamics, particularly migration. The social science literature on migration resulting from environmental degradation, including forest degradation, is robust and well-documented (42), as is the literature on the impact of migration on environmental conditions in migrant destinations (43, 44). There is emerging recognition that outmigration from origin communities might change demand for land and lead to FR (45, 46). However, there is debate about causality and the nature of the impacts (47, 48). Consider Thailand, where a land-use transition from annual crops (rice, maize) to longer-term crops (rubber, fast-growing trees) has been reported (49). Some research indicates that this transition has resulted from local labor resources migrating for longer stints (50) or from migrants bringing back social remittances (51) that reshape land-use behaviors (52). Others argue that government investments in FR projects have displaced populations (49).

We organize the state of knowledge on demographic aspects of FR into three sections. First, we recap classic formulations of how demography impacts forest cover, through spatial extensification of livelihoods resulting from population pressure or outmigration relieving pressure on natural resources. Second, we discuss modifications of the classic formulations, including recent findings and arguments about how sustained migrant ties to areas through circular migration or financial and social remittances might affect FR variably, either working in concert with policy interventions or contravening them. Third, we discuss how FR programs may or may not lead to population stability and reduced population pressure on resources.

### 4.1. Classic Formulations of Migration and Forest Degradation

Malthusian theories about human behavior in relation to natural resources, especially resources in the public domain, are at the root of many predictions about migration and forest cover (53). Combined with evolutionary and archaeological ideas about how environmental endowments pull people to places and yield settlements and population growth (54, 55), these formulations argue that population growth is associated with resource extraction and land-use change, often environmental degradation. Frontier settlement research throughout the 1990s and into the present provides evidence on migration into forested regions leading to forest degradation (55).

The flipside of this argument is that resource constraints and environmental degradation will lead to demographic decline through some combination of changes in demographic outcomes, such as increased mortality, lowered fertility, and migration out of locales with remaining resource endowments too poor to support livelihoods (56). Although this demographic decline may relieve population pressure on resources, more often than not it yields vicious cycle models of negative feedback loops between poverty, demographic vulnerability, and resource degradation (56). Evidentiary support for these generalized patterns is substantial (48).

Growing evidence suggests that Malthusian explanations are incomplete, however. Household dynamics create heterogeneity in the number of households and their environmental impacts (57). Households may divide into two as they grow, one with members at an earlier life cycle stage and the other with members at a later stage. Each household will burden resources in different ways, making generalization about the effect of household size on the environment difficult. Similarly, households may pursue sequential moves across a frontier landscape after an initial move into a frontier region, and each type of move could have different environmental effects (57).

Numerous studies have also shown how households might stay in a locality but employ migration in numerous ways, including by sending members away to work through short- and long-distance moves, practicing circular migration, and receiving remittances. Once households are settled in frontier areas, or in close proximity to forest lands, migration out of the region can diversify incomes and smooth income flows (58). However, outmigration might not relieve pressure on resources. Extensification into forested land may continue through the planting of upland crops, which might be supported by remittances and available household labor (59). In addition, migrant networks to distant markets can increase pressures on other resources by shifting local resource extraction toward demands generated by migrant-facilitated markets (60–62).

Some suggest that land cover change from annual cropping to longer-term crops, including tree plantations, occurs when local labor resources migrate for longer stints, reducing the availability of labor and favoring more permanent types of crops that require less labor (45). Much of the forest transition literature follows this line of reasoning, arguing that rural-to-urban migration can relieve pressure on both private and communal lands and lead to a return to forest cover, either by design or naturally. A rare impact evaluation of migration’s influence on FR finds that outmigration from rural areas in Nepal for guest-worker jobs abroad increased tree cover, especially on flatter land where labor-saving farming technologies are more feasible (46). Most studies have investigated the impacts of domestic migration and not used impact-evaluation methods. Examples include studies reporting that rural-urban migration increased FR in the Atlantic Forest in Brazil (63) and explains greening trends in the Southwest China Karst (64) and across mountainous regions in China (65).

However, a study in the Atlantic Forest of Argentina finds mixed results after examining 30 years of land-use change and demographic pressures (66). It questions the utility of assuming that migration is a pressure-release valve. A study of all municipalities in Mexico finds little impact of demographic change on land cover during 2001–2010 (61). A comprehensive mapping of forest cover change in Latin America and the Caribbean finds considerable variation, and it speculates that land demand for food requirements offsets factors that favor FR, including outmigration (67).

A recent study in Mexico (68) suggests that not only rural-urban migration but also livelihood diversification pathways (56) can lead to a forest transition. These pathways include changes in technology, changes in agricultural practices, and declines in the use of forest materials for housing and energy in both proximate and distal locales. Additionally, the type of migration from a rural area influences impacts on proximate forests. An examination of 2001–2010 forest cover change in Mexico finds that circular migration appears to be associated with deforestation, while, as in the Nepal study (46), international migration that is of longer duration and supplies substantial financial remittances to origin communities appears to best explain recovery of coniferous and dry broadleaf forests (69).

### 4.2. Demography Alone Does Not Determine Forest Degradation or Recovery

These mixed patterns of influence argue for a more comprehensive framework in assessing the multiscalar, spatial and temporal dynamics between migration and forest cover. There is good reason to seek explanations that integrate complex mechanisms—namely, micro and macro contexts that govern social conditions, economics, and cultural institutions—that modify demographic behavior and moderate its impact on the environment. Unfortunately, this line of research is not yet well-developed in terms of a focus on forest cover. Moreover, ascertaining causality is a persistent challenge for most analyses within this line of research, which rarely use impact evaluations due to the relatively long time horizon for observing migration effects and forest cover change. Some suggestive findings can nevertheless be drawn from the literature.

Migrants’ impacts on the environment depend on how they arrive and are incorporated into destination communities and how their departure rearranges institutions in their origin communities (70). Arriving migrants’ access to local social capital—the combination of cultural and social resources that facilitate their assimilation and incorporation within a destination community—is one mechanism explaining their variable environmental impacts. For example, migrants arriving in coastal areas of Indonesia through marriage networks practice more sustainable fishing practices than do migrants with few socio-cultural ties connecting them to local livelihood practices and lifestyles (43).

A comparison of Mexico and India illustrates how rural-urban migration can rearrange social institutions, organizations, and relations in origin communities (70). In both countries, traditional, communally managed resources, whether forests or fisheries, are less well-managed with growing migration out of a region. Other research argues, however, that this pattern is partly explained by communal resources becoming increasingly managed privately, which in turn propels migration (71). That is, migration might be a consequence, not a cause, of rearranged institutions.

A consistent finding is that movement out of a place is not always permanent and may involve ongoing ties through return migration, circular migration, and remittances. While a migrant remains tied to a place, the act of migration can disrupt and reorganize social relationships to landscapes. A historical analysis of outmigration from, and then return migration to, St. Lucia shows how shifting settlements, economic development, and lifestyles influenced relationships to landscapes, including FR in mountainous regions, intensified forest loss in coastal areas, and forest management in a few concentrated areas (72).

Migrants bring back to their origin communities not only financial remittances but also social remittances, which can lead to investments in more sustainable natural resource practices (73, 74). Migration in LMICs is frequently understood within a livelihoods framework, which identifies and recognizes a multiscalar set of contexts that shape household decisions in relation to environmental endowments. In this framework, financial remittances can combine with social remittances to change the relationship of rural communities to forests (75). In Indonesia, female migrant remittances sent home to husbands and sons have shifted livelihoods and reduced forest degradation pressures by stimulating investment in new crops grown in the forest understory (76). A review of the Kenyan literature on rural-urban migration and the environment calls for a more nuanced assessment of household livelihood strategies (77). Such an assessment would account for households’ locations along a continuum of commodification and subsistence, in a context of trans-local networks that transmit people, identities, resources, and, importantly, information.

Fully accounting for the many different types of migration, including nonunidirectional mobility, is a crucial next step in research on migration and FR. The migration literature has shown that moves are not singular, not always permanent, often circular, and sometimes sequential (44). Migration as one response to changing conditions must also be considered in relation to immobility: staying in place. Explanations for immobility are not solely the inverse of explanations for migration. Far more people, on the whole, are immobile than mobile. Future demographic research on FR should adopt a comprehensive approach to understanding the entire gradient from immobility to mobility (78).

### 4.3. Forest Restoration Interventions and Stabilizing Population Growth and Settlement

Programs to establish conservation areas or restore forests have generated insights on how such programs might be associated with involuntary population displacement, voluntary outmigration, or population stabilization. As noted earlier, studies in Thailand argue that government reforestation projects have displaced settlements (49). Government-sanctioned commercial tree-planting projects displacing communities holding customary land rights have also been reported in Indonesia and Malaysia (79).

Studies on China’s SLCP provide the most extensive evidence on FR programs’ impacts on outmigration. China launched the SLCP in 1999 in response to catastrophic flooding. By 2015, the national government had paid US$69 billion to 32 million smallholder farming households in 25 Chinese provinces to restore forests on 15 Mha of sloped cropland (4). Payments end after 16 years. The SLCP has induced households in many locations to shift their income sources first from crops to livestock and then to off-farm employment (80). Impact-evaluation studies in two provinces, Anhui (81) and Ningxia (82), report large, significant impacts on outmigration, with households in Anhui using outmigration as a primary means of diversifying income sources in anticipation of the end of the payments. This livelihood diversification strategy reportedly reduced poverty (83).

Limited but promising evidence suggests that linking conservation, FR, and a livelihoods framework approach can stabilize rural populations and minimize population pressures on forests (84–86). However, these outcomes require significant investments and are not certain (87, 88). Similarly, case studies from Mexico (89), Indonesia (90), and Ethiopia (91) indicate that migration and forest cover change, including FR, can coincide with lifestyle changes that are mutually reinforcing, but this literature has not been systematically and scientifically evaluated in relation to migration or population settlement.

## 5. FOREST RESTORATION METHODS

The characteristics of forests restored in response to market forces, policy interventions, demographic dynamics, and other factors depend on the restoration methods applied by landholders. In turn, the technical feasibility of these methods depends on site conditions shaped by soils, climate, and land-use history. In this section, we draw on the ecological and silvicultural literatures to characterize the major processes of ecological degradation resulting from forest clearance for other land uses, and we describe alternative FR methods and present evidence on their effectiveness. We focus on restoration of forestland that has been cleared for agriculture, which can be thought of as a one-time conversion process that causes medium-severity degradation. Despite this focus on afforestation, many of the FR methods described apply to situations of reforestation too.

### 5.1. Characterizing Degradation

Characterizing degradation is necessary for developing logical and feasible methods of restoration. Large regional differences in agricultural land conversion exist and can be categorized as being for either large-scale, export-oriented commodity production or small-scale farming for subsistence or domestic markets (92). The chief difference between the two categories is the intensity, efficiency, and scale at which conversion takes place. Conversion for large-scale production of commodity products is intensive, removing all vestiges of the original vegetation and often modifying the contours of the landscape to enable mechanized access for treatments to the soil and crops (93). In contrast, smallholder conversion involves a diverse set of agents ranging from indigenous communities to sedentary private small landowners and migratory, often landless peasants (94–96). It tends to be incomplete, leaving vestiges of the original forest behind either purposely, because many trees and shrubs have economic values, or due to inefficiencies in the clearing process itself. Conversion is incomplete chiefly because smallholders have less access to capital and labor compared to larger industrial enterprises (97). Remote sensing studies can mischaracterize such conversion as permanent when in fact much of the cleared area might return to forest under shifting fallow or swidden agricultural systems (96, 98). The Supplemental Appendix provides more details on the impacts of forest clearance for agriculture on native vegetation and soils.

### 5.2. Characterizing Restoration

Two broad approaches can be taken with restoration, passive restoration and active restoration. Passive restoration is entirely dependent on in situ living vegetation, a seed source within the soil, or seed that is distributed from nearby vegetation by animals, wind, or water (93). No planting, seeding, or site preparation is necessary, although fencing or other protective measures might be required. Passive restoration is inexpensive, and it provides a range of ecosystem services that are often hard to monetize (e.g., watershed stabilization and erosion control, increased soil productivity, biodiversity).

Active restoration is more expensive, requiring more capital and infrastructure along with more silvicultural knowledge (93, 99), but it can accelerate FR and shorten time to canopy closure. Reasons for active restoration are either the infeasibility of passive restoration due to the lack of seed or a vegetative source or the ability of active restoration to supply higher levels of desired goods and services in less time. Active restoration methods differ according to the vegetation succession model they are based on, either initial floristics or relay floristics.

#### 5.2.1. Passive restoration.

The most obvious example of passive restoration is forest coming back naturally on smallholder lands that have gone out of agricultural production. The forest that comes back in such situations has been termed secondary forest of old-field origin (100).

Much of this type of natural regeneration has been reported on pasturelands in the Neotropics (100). In general, aboveground biomass recovery during Neotropical secondary forest succession is highly productive and resilient, but it varies 11-fold across sites after 20 years and takes nearly 70 years to recover to 90% of old-growth forest levels (101). A study in Panama showed that soil carbon and aboveground biomass have distinct relationships with stand age and soil fertility, with soil carbon recovering within 40 years from the start of regeneration but aboveground biomass continuing to increase past 100 years (102). Secondary forests in the Amazon recover remarkably fast in species richness (number of species) but slowly in species composition (relative abundances of different species) (103). They take a median of five decades to recover the species richness of the original old-growth forest, whereas full recovery of species composition takes centuries.

Forests in Central America and Africa evidently recover faster from past disturbances than those in South America and Asia (104). A dearth of studies from Asia and Africa makes this generalization tenuous, however.

#### 5.2.2. Active restoration using initial floristics.

Direct seeding of open areas is perhaps the simplest and least-cost active restoration method if the purpose is quick revegetation for land conservation and the choice of particular species is less important. It is most appropriate when soils are easy to prepare, seeds of appropriate native vegetation can be gathered efficiently, and seed predators are low or absent (93). Such circumstances are unusual, and only a few studies on direct seeding exist (105, 106). One example is a study from Cerrado, Brazil, where old arable land can be plowed easily and labor to collect seeds from nearby forest fragments is relatively cheap (107). After four years, direct-seeded sites had high aboveground biomass, had formed a multilayered canopy, and were starting to be colonized by nonseeded species. Another example comes from Lao PDR, where direct seeding seems possible on old agricultural soils provided that species-site matching is well-defined and seeds are buried to reduce desiccation and predation (108).

The planting of a relatively select list of pantropical pulpwood and sawtimber species, e.g., *Acacia*, *Eucalyptus*, *Pinus*, and *Populus*, in single-species, single-aged plantation systems is the most widespread application of active restoration in LMICs. These species have been widely planted for the past 60 years, usually on old agricultural fields in countries where they are not native. The planting objective is income from repeated wood harvests, not ecosystem restoration per se. The conversion of degraded agricultural sites to timber plantations has many potential ecological benefits, including soil remediation, watershed stabilization, and carbon sequestration (109), but it usually does not provide high conservation or wildlife habitat values (but see Section 5.2.3).

Forest plantations have expanded at large scales in South America (e.g., Brazil, Chile, Uruguay) and Asia (e.g., China, Indonesia, Vietnam) (15, 93, 110). Their success is attributable to multiple factors: The selected plantation trees are fast-growing and tolerant of poor soils, droughts, or fires; have easy and known modes of seed collection, storage, and propagation; have been the target of tree improvement programs; and have well-established global wood markets (93). Many additional species might be suitable for forest plantations but more compatible with restoration for conservation purposes because they are native to a region (111–113). Insufficient knowledge on seed collection, storage, and propagation and growth and yield for these species inhibits their acceptance by landholders and investors, and so does poor market infrastructure for their wood (109, 112).

Although most forest plantations in LMICs have a single species, there can be multiple reasons to plant mixtures of species: increased resiliency to pests and pathogens compared to mono-dominant plantings; increased net primary productivity; and a greater diversity of products (114). Ecological theory provides the basis for many of these potential advantages, as negative density-dependent processes can reduce insect and disease mortality, and complementary interactions can increase productivity. Few studies have demonstrated these advantages empirically. A study in Panama suggested positive effects of negative density dependence, as relative productivity increased with increased species richness in tree plantings (115). A study in Queensland, Australia, similarly demonstrated that basal area increased with tree-planting species richness (116).

Complementary interactions have been well-documented in succession theory for native forest development and within the silvicultural subdiscipline of stand dynamics (117), but few studies have demonstrated them well in forest plantation applications (114). The main mechanisms relate to differences among species in phenology, with deciduous species comprising an overstory above more shade-intolerant evergreen species, which can assimilate carbon at times of the year when the deciduous species have shed their leaves; rooting, with shade-intolerant overstory tree species having a deeper root system than the more lateral/superficial rooting system of the shade-tolerant understory, thereby reducing competition for water uptake (118); and successional status, with shade-tolerant species being longer-lived than shade-intolerant species provided there is no disturbance (114).

Compared to monocultures, mixed-species plantations are expected to increase resiliency to market fluctuations thanks to their greater diversity of products, but they are more complex to design and manage (119). Empirical evidence on the economics of such plantations is even more limited than evidence on their ecology, with few studies covering entire management cycles (119).

#### 5.2.3. Active restoration using relay floristics.

Birds generally visit larger trees, promoting the greatest density of seed rain below and facilitating subsequent understory tree seedling establishment (111). This observation has led to studies on FR via nucleation: using individual, isolated plantings to facilitate natural seeding and expansion into open spaces. Studies in Australia showed that living trees promoted the colonization process more effectively than bird perches, but colonization was modest, and a couple of native species dominated species composition (120). Studies in old pastures in Costa Rica demonstrated the effectiveness of using either existing trees and shrubs or purposeful tree plantings in groups to facilitate natural regeneration through seed rain, primarily dispersed by birds (121, 122).

Another approach is to use timber plantations as old-field conifer analogs. Following historical agricultural abandonment in many temperate regions, one species, usually a conifer, colonized open fields, shading out grasses and facilitating seed dispersal by birds and arboreal mammals and the establishment of more shade-tolerant native broadleaf species beneath. De facto, many commercial plantations in the tropics have similarly facilitated the establishment and growth of shade-tolerant native forest trees beneath the non-native timber species (123, 124). In addition, studies have successfully planted and then released rainforest species of timber, nontimber, and conservation value within forest plantations (125). This approach merits greater consideration, as commercial forest plantations in LMICs and their potential for conversion to native forests and mixed plantings dwarf governmental and nongovernmental FR initiatives based on direct seeding or planting of native trees in the open. In fact, Vietnam has implemented a pilot program whereby smallholders receive a one-time subsidy for planting shade-tolerant native tree species under the canopy of exotic timber species (126).

### 5.3. Establishment Costs

Foresters refer to the costs of activities required to implement the FR methods described above as establishment costs. Tree-planting costs (seedlings, labor, etc.) are a prime example, but even passive restoration can involve costs, such as fencing (127). Data on establishment costs in LMICs are very limited. For example, the natural climate solutions study (39) used a uniform establishment cost for the entire world drawn from an earlier study (128), which in turn used data that were mainly from the 1990s and included few LMICs. A rare recent survey of actual FR projects found great variation in establishment costs for both passive and active restoration across three biomes in Brazil (129). Similarly, large variation has been reported for tree-planting costs in a global sample of countries, including several LMICs (130).

As expected, the Brazil survey found that active restoration was much more costly than passive restoration. However, it also reported that landholders more commonly use active restoration than passive restoration. Landholders likely prefer the more costly method because it generates higher returns in time-adjusted terms (27): Active restoration may cost more up front than passive restoration, but its tendency to restore forests more rapidly (131), especially in the tropics (132), boosts the present value of the benefits it generates. Active restoration can also be superior to passive restoration because it allows for more predictable species composition and better growth form of trees (93, 133). Passive restoration is attracting increased attention as a low-cost FR approach, especially for carbon sequestration (134), but its merits relative to active restoration depend on the rate at which it generates benefits, whether market (e.g., wood production) or nonmarket (e.g., carbon sequestration), not merely its cost.

Cost is an incomplete measure of the relative merits of different FR options for a second reason too: It does not account for the risk of conversion back to nonforest land uses. This re-deforestation risk appears to be higher for naturally regenerated secondary forests (135, 136) than for planted forests (137, 138) in LMICs. Although regional factors could explain the observed difference in re-deforestation—the data on secondary forests come from Latin America, whereas the data on planted forests come from Asia—the difference is not surprising: The lack of active restoration on naturally regenerated sites could reflect landholders’ determination that forests do not generate significantly higher returns than nonforest uses of those sites, in which case even a small increase in nonforest returns could induce re-deforestation of secondary forests. Assessments of the projected benefits of different FR options need to account for the likelihood that restored forests will persist.

## 6. INTERVENTIONS TO PROMOTE FOREST RESTORATION

As the foregoing discussion indicates, landholders weigh expected benefits against opportunity and establishment costs when deciding whether to adopt FR methods. We now review three interventions that can shift the balance in favor of adoption. The first is a purely private-sector mechanism: outgrower schemes. A long-standing research finding is that households with smaller land plots are less likely to plant trees (139). Outgrower schemes offer smallholders financial and technical support to overcome obstacles that discourage them from planting trees to supply wood markets.

The remaining two interventions are government actions. One is to strengthen land rights. The rationale for stronger land rights promoting FR is simple: FR is a form of investment, and stronger land rights increase landholders’ confidence that they will reap its returns. Households with more secure land rights have long been identified as being more likely to plant trees than households with less secure rights (12, 139). The final intervention is payments for ecosystem services (PES) (11, 140). In contrast to the first two interventions, PES programs can target restoration of types of forests (e.g., mixed native species) that provide nontimber services that markets do not reward landholders to supply.

These three interventions are not a comprehensive list. For example, information campaigns based on farmer-to-farmer study visits were a major factor in the widespread adoption of farmer-managed natural regeneration of trees on agricultural land in Niger after the mid-1980s (141). Some of the examples presented below provide additional evidence on information’s role in FR. We also note that interventions can interact. For example, smallholders with stronger land rights are more able to participate in outgrower schemes (142, 143).

### 6.1. Outgrower Schemes

Small, fragmented landholdings discourage the establishment of wood-processing facilities by making wood supply more costly and less secure for forest products companies. To overcome this obstacle, companies in various LMICs, including Brazil, Colombia, Ghana, India, Indonesia, the Philippines, the Solomon Islands, South Africa, Thailand, Vanuatu, and Zimbabwe, have recruited smallholders as wood suppliers through outgrower schemes and other partnership programs (142–144). These programs can involve tens of thousands of smallholders with planting areas as small as 1–2 ha (142). Companies offer outgrowers technical support, a guaranteed market, and sometimes advance payments. Without the technical support, smallholders might lack information on which tree species to plant and how to cultivate them. Through these various forms of assistance, smallholders can gain an income stream that is less risky than if they grow wood independently.

However, smallholders might not be able to negotiate contracts that offer them fair terms, especially with respect to the market guarantee. Interviews with timber outgrowers in Indonesia suggest that the issue is not whether outgrowers benefit at all from the schemes, but whether they benefit as much as they could: Although 93% of outgrowers reported that they had benefitted economically, 73% did not fully understand their contracts, and 89% did not know they could renegotiate the contracts and the prices companies paid them (145).

Discrete-choice experiments (DCEs) reveal that smallholders’ preferences for the design of outgrower schemes can vary substantially, even within the same village. A DCE in Indonesia investigated preferences related to six features of a hypothetical scheme intended to promote pulpwood growing on private smallholdings: contract length, required labor inputs, income received, timber production insurance, training, and local road improvements (146). It concluded that the scheme would be more likely to succeed if it offered three types of contracts with different primary objectives: wood production, conservation, or livelihood development. Although this finding is specific to outgrower schemes, it signals the general importance of engaging landholders in the design of FR programs to ensure that the programs align with their preferences (147).

### 6.2. Strengthening Land Rights

The relationship between FR and land rights is complex. The most obvious source of complexity is that statutory (legal or formal) rights vary between and within countries and do not necessarily coincide with customary (informal) rights. Governments owned most forestland in LMICs in 2015 (70%), but regional shares ranged from 37% (Central America) to 99% (Western and Central Asia) (15). Ownership by private parties—individuals, communities, and businesses and nongovernmental institutions—rose during 1990–2015, as did management of government forests by private parties. Private ownership or management by communities has increased in some countries, but communities’ customary rights remain incompletely recognized in many others (148). Research on the impacts of private land rights on forestland use in LMICs has focused more on community rights than individual rights (149). Understanding the impacts of individual rights is essential too, given the importance of marginal agricultural land as a historical and potential site for FR. In contrast to forestland, most agricultural land, especially cropland, in LMICs is owned or customarily held by individuals and businesses, not by communities (150).

An additional source of complexity is that rights to trees can differ from rights to the land they grow on. Onerous regulations on harvesting, transporting, and processing trees grown on private land can impede landholder investments in tree growing (141) and community investments in forest enterprises that communities need to earn income from their trees (151). Numerous other factors mediate the relationship between land rights and landholder investment, including land use, markets, social norms and practices, and governance (9). Existing FR planning tools provide only limited guidance on assessing these various sources of complexity (152).

Land-rights interventions have taken various forms. Common ones include strengthening customary rights, for example by demarcating, documenting, and legally recognizing lands held under these rights, and converting communal land to separate parcels that have individual ownership or use rights (9). A recent systematic review of these interventions on agricultural land identified only 20 quantitative and nine qualitative studies that satisfied its methodological screening criteria (9). The review did not focus specifically on FR, but it did include three quantitative studies that analyzed impacts on tree planting. All three found that land-rights interventions increased planting. An overall finding of the review, however, was that determining the general impacts of land-rights interventions on smallholder investments, including investments in trees, is difficult due to methodological problems with existing studies and the impacts being mediated by multiple factors.

The review noted that none of the reviewed studies were randomized controlled trials (RCTs). Since its publication, RCTs in Benin (153) and Zambia (154) have tested the impacts of strengthening customary use rights on tree planting. Both RCTs randomly assigned a community mapping intervention, which demarcated farmers’ land parcels, across treatment and control villages. The intervention significantly increased perceived security of land rights in both countries, but it had no impact on tree planting in Zambia, and its impact in Benin was approximately an order of magnitude smaller than impacts reported by previous studies on land rights and smallholder investment. The authors of the Zambia study argue that the larger impacts reported by previous studies could be due to methodological problems with those studies, and they support this argument by demonstrating that the methods used in those studies yield positive, significant impacts when applied to the Zambia data. The design of the RCTs might be part of the explanation too, however: The RCTs measured very short-run impacts, within only approximately a year after the intervention. Impacts might rise as time passes. Still, the RCT results raise the possibility that strengthening customary rights might not increase FR as much as results from earlier studies implied.

The Zambia RCT had a more complex design than the one in Benin, as it also included an intervention that provided farmers with free seedlings, training, and technical support at no cost. In contrast to the land-rights intervention, this financial cum informational intervention had a significant, large, positive effect on tree planting. In effect, the latter intervention provided farmers with the types of support that companies provide smallholders under outgrower schemes.

Evidence that land-rights interventions can promote FR is stronger for the conversion of communal land to parcels with individual use rights. [Evidence on the FR impacts of interventions that privatize land to communities and associations (155) is scarce.] Vietnam’s 1993 land law effectively privatized much communal rural land, including degraded hilly forestland. The law and its subsequent revisions in 1998, 2000, and 2003 provided legal nonstate entities, including individual households, with long-term (50-year) land-use certificates (Red Books) that could be sold, leased, mortgaged, and inherited (156). The privatization occurred in two steps, with the government first allocating land to households and subsequently issuing formal land titles. An impact evaluation found that both steps increased tree planting, but the second step increased it much more (157).

China’s forest tenure reforms began in 1981, when the government introduced two tenure regimes: family forestland, which was allocated to households for planting trees for their own use and could be inherited, and household responsibility system forestland, under which households received 5–15-year management contracts for collective forestland (158, 159). Starting in the late 1990s, a series of reforms expanded households’ rights over family forestland, e.g., by allowing land transfers and use of land as collateral, and made contracts on household responsibility forestland renewable for 70-year terms. Impact evaluations have found that these reforms significantly increased household investment in the establishment and management of planted forests on family forestland within just a few years of their implementation (158) and that the reforms’ overall positive impact on FR persisted beyond the initial years on both family forestland and household responsibility forestland (159).

### 6.3. Payments for Ecosystem Services Programs

Research on FR PES programs spans a range of topics, including program design, participation, cost-effectiveness, and socioeconomic impacts. Experimental approaches—DCEs, RCTs, and pilot auctions—dominate research on program design, with a focus on tree-planting contracts on agricultural land. All DCEs on FR PES programs include the payment to the landholder as one contract attribute, but they tailor the other attributes to local conditions. For example, additional attributes in a DCE in Panama included contract length, tree insurance, and penalties for non-compliance (160). DCE results confirm that these other attributes matter, i.e., that landholders care about more than just the payment level. A DCE on China’s SLCP included two attributes related to land rights: the right to rent land enrolled in the program, and the risk that the government would redistribute farmers’ land. It found that reforms that granted the former right and eliminated the latter risk would induce farmers to continue to participate in the SLCP after its current phase ended, even if the government reduced the payment level (161). Designing contracts to align with landholder preferences does not necessarily ensure that PES programs can scale up FR, however. Results of the Panama DCE imply that an efficiently designed FR PES program would induce landholders to enroll only 2% of the nonforested, nonurban private land in the Panama Canal watershed (160).

RCTs involving private smallholders in Uganda (162) and Zambia (163) have investigated the impacts of payments linked to short-run seedling survival. In Uganda, the payments significantly increased not only survival but also the share of households that planted trees and the mean area and mean number of trees they planted. The Zambia RCT had a more complex design, which offered farmers payments at two times: an initial subsidy for purchasing seedlings and a later cash prize if a minimum number of seedlings survived. The subsidy increased seedling purchases but did not affect survival, whereas the prize increased survival. These results suggest a budgetary tradeoff in scaling up FR via PES programs: An implementing agency can boost landholder participation by allocating more of its budget to subsidize seedling purchases, but boosting tree survival requires allocating more of the budget toward payments linked to survival.

Operational PES programs in LMICs offer landholders fixed payments, which might vary according to predetermined criteria but are the same for all landholders who meet those criteria. Auctions offer an alternative payment mechanism, with payments flowing to the landholders who bid the lowest amounts (i.e., request the least compensation) to modify their land-management practices (e.g., to plant trees). In theory, auctions can be more cost-effective than fixed-payment PES programs, but their cost-effectiveness depends on their design features, in particular their pricing rule—do winning bidders, of which there can be many in a single auction, receive payments based on their individual bids, or do they all receive the same, auction-clearing payment?—and transactions costs, which can be high (164). Experience with FR auctions in LMICs is limited to a handful of pilot applications (164). Auctions in China (165) and Malawi (166) offer evidence of small to medium gains in cost-effectiveness. An auction in Tanzania suggests a tradeoff between cost-effectiveness and poverty alleviation: tree-planting payments flowed mainly to better-off households, who were more likely than poorer households to be the winning (i.e., low) bidders (167). However, females tended to bid lower than males in that auction and under certain auction designs in Kenya (168), which suggests that FR auctions can be pro-gender if not pro-poor.

Studies on the socioeconomic impacts of FR PES programs have mainly investigated impacts on household income (11, 12). If landholders are well-informed and participate voluntarily in PES programs, then one would not expect participation to negatively affect them. Landholders do not have perfect foresight, however, and they might not understand tree-planting technologies well (163). Both reasons could cause them to lose income even if they participate voluntarily in FR PES programs.

Evidence on the income effects of FR PES programs refers mainly to China’s SLCP (11). Three features of the SLCP suggest that it might not increase the income of the average participating household: The Chinese government set payments at levels intended to compensate households for the average value of lost crop production from enrolled plots, not to provide any surplus above that amount; the program called for households to receive payments for a limited period, not permanently; and participation was not voluntary in all locations (34). Consistent with these features, impact evaluations generally report that the SLCP has not significantly affected total household income (34, 81, 169, 170).

## 7. SCALING UP RESTORATION

Large-scale FR will occur in LMICs only if millions of landholders expect FR’s benefits to exceed its costs. Landholders do not necessarily need to receive benefits in monetary form. FR can generate goods and services that contribute to household consumption and welfare directly (e.g., fuelwood supply) or indirectly (e.g., increased yields of subsistence crops). With the aggregate cost of restoring 0.35 Bha of forestland during 2021–2030 estimated at US$1 trillion (171), however, most landholders will surely invest in FR only if they benefit monetarily.

Section 3 examined two potentially scalable sources of monetary benefits. One was income from growing wood for commercial products. This source appears unlikely to deliver much FR without expanded use of wood beyond conventional products. Expanding wood use requires a combination of R&D and sustainable value chains linking landholders to markets for new wood products (172). The other source was carbon payments. The studies reviewed in Section 3 predict that carbon prices in the range of US$80–100/ton CO_2_e would make large-scale FR economically feasible. A huge gap exists between this range and the current average global carbon price, US$2/ton CO_2_e (173). Even closing this gap might not be sufficient to achieve the Bonn Challenge goal by 2030 (41). Carbon payments as an incentive for large-scale FR require a more aggressive global policy response to climate change, one that generates much higher carbon prices.

Increased monetary flows from expanded wood use or higher carbon prices might enable LMICs to attract significant new sources of private capital for FR. Pension funds, insurance companies, and other financial institutions appear to be especially promising sources. These institutions already invest globally in forestland for wood and, increasingly, monetized nontimber benefits (130, 174). Macroeconomic and institutional factors currently limit their forestland investments to only a few LMICs, however (130, 175).

Government-funded PES programs are a third potentially scalable source of monetary benefits. China’s SLCP is the leading example of this approach. Record government debt in LMICs caused by the COVID-19 (coronavirus disease 2019) pandemic (176) has dimmed the near-term prospects for new, large-scale PES programs in other countries, but economic recovery strategies could perhaps expand the role of FR in employment-based social assistance programs (177). The near-term prospects for foreign assistance supplementing LMIC government funding of FR have dimmed too, as the pandemic has also affected budgets in donor countries. Moreover, total foreign aid focused on the environment in the agricultural, forestry, and fishing sector was only US$4 billion/year during 2016–2017 (178), which is tiny compared to FR’s estimated US$100 billion annual cost during 2021–2030.

A fourth potentially scalable source is through legal FR requirements, in which case landholders’ “benefits” come from avoiding sanctions on noncompliance. An example is Brazil’s 2012 Native Vegetation Protection Law, which requires private landholders to restore their land to achieve a minimum percentage area of native vegetation (5). The law has faced a series of implementation challenges, however, and imposing similar legal requirements in other LMICs is unlikely to be politically viable until economies recover from the pandemic.

Scaling up FR thus faces great challenges. These circumstances elevate the importance of using the land, labor, and capital available for FR efficiently in pursuit of the social and environmental objectives of FR initiatives. Efficient use means maximizing the expected net benefits from FR, not maximizing the area restored. Stating that resource-use efficiency is important for FR is easy. Realizing it is much more complex, with details varying by location and FR initiative. In all cases, however, FR initiatives are more likely to yield a maximum return on the scarce resources they require if their planning and implementation are well-informed by knowledge on both biophysical and socioeconomic aspects of FR.

## Supplementary Material

Appendix

## SUMMARY POINTS

1. Information on biophysical aspects of forest restoration (FR) in low- and middle-income countries (LMICs) is more abundant than information on socioeconomic aspects, because social science research on forests in LMICs has focused more on reduced deforestation than on FR.
2. Although trends suggest that forest transitions are underway in many LMICs, realizing the FR pledges that governments have made in the Bonn Challenge and other international FR initiatives will require a massive scaling up of forest restoration.
3. Increased wood demand combined with policies that reward forest carbon sequestration could propel substantial FR in LMICs; the economic incentives thus provided to landholders are important given continued competition for land with agriculture and pandemic-constrained budgets in LMICs and aid agencies.
4. Research findings are inconclusive on (*a*) migration’s impacts on forest cover, including on FR, and (*b*) the impacts of FR efforts on migration. The mixed findings are partly due to varying ecological and social conditions across case studies but also to a lack of systematic accounting for the variety of migration types and the multiscalar nature of migration effects along both temporal and spatial dimensions.
5. Wood plantations often create silvicultural conditions that foster natural regeneration of native species in their understory, which offers a potentially large opportunity to convert plantations over time to biologically richer forests that provide ecosystem services that plantations do not.
6. Natural regeneration of secondary forests on old fields can restore and conserve native vegetation where viable ecological conditions occur, but the persistence of secondary forests depends on landholders deriving sufficient value from them.
7. Strengthening customary land rights might not promote as much FR as earlier research suggested it would, but converting communal land to individual parcels has contributed to large-scale FR in some countries (e.g., China and Vietnam).
8. At least one payments for ecosystem services (PES) program has achieved large-scale FR (China’s Sloping Lands Conversion Program); evidence from this and other forest-restoration PES programs indicates that landholder participation depends on more than just the payments they receive.

## FUTURE ISSUES

1. The slower expansion of planted forests during 2010–2020 compared to 2000–2010 warrants investigation, as it might indicate unforeseen social and ecological limits to achieving forest restoration (FR) goals.
2. Research on many aspects of FR, including its relationship with migration and interventions intended to promote it, would benefit from the creation of new household and land-use datasets that span longer time periods, with sufficient spatial resolution and scale to enable integrated investigation of short-run and long-run processes that occur over varying distances.
3. Few studies have demonstrated the productivity and resiliency advantages of mixed-species plantations compared to monocultures; more ecological experiments are needed to test these potential advantages so as to develop silvicultural guidelines for establishing and managing such plantations.
4. A crucial next step for research on migration and FR is to account for the full array of types of migration, from permanent moves to temporary ones, sequential moves, and circular migration, and the entire gradient from immobility to mobility, given that far more people stay in place than move.
5. Future research on migration and FR should study the transmission and diffusion of ideas through migrant networks, which can shape the valuation of environmental endowments and human-environment relations among individuals and within households; collaborations among ecologists, anthropologists, demographers, and social or environmental psychologists could prove valuable in this research.
6. Evaluating the potentially negative impact of large-scale FR on food security requires the development of economic models of global wood and food markets that are based on higher resolution data, allow for greater spatial variation in the effects of forestry and agricultural returns on land-use change, and incorporate migration’s effects on land use.
7. Expanded use of impact evaluation methods is needed to generate unbiased estimates of the social and environmental effects of existing FR interventions, especially interventions that promote FR to supply wood, sequester carbon, or both; these evaluations should encompass a representative range of locations, with examples of both passive and active restoration, and should measure distributional impacts (in particular, on vulnerable groups) and not just the average impact across all landholders.
8. Where possible, the design of new FR interventions should be informed by social science experiments (e.g., discrete-choice experiments, randomized controlled trials, pilot auctions), which enable researchers to test alternative designs and determine how to accommodate variation in landholders’ preferences related to land use and the interventions’ features.

## Abbreviations

Forest: an area of >0.5 ha with trees >5 m high and canopy cover >10%; not predominantly in urban or agricultural use
Afforestation: establishing a forest on land that might have originally been a forest but is currently in a nonforestry use
Reforestation: establishing a forest on land where forestry is the current use but tree cover was recently lost
Low- and middle-income countries (LMICs): countries with gross national income per capita below World Bank–determined thresholds in most years during 2000–2020
Impact evaluation methods: a set of quantitative social science methods, including randomized controlled trials, difference-in-difference models, instrumental variables, regression discontinuity, and matching methods
Planted forest: plantations and other forests with planted or deliberately seeded trees comprising more than half of mature growing stock
Active restoration: restoration by planting seedlings, sowing seeds, or site manipulation (e.g., soil scarification) after controlling disturbances that inhibit tree survival
Passive restoration: restoration through natural regeneration without planting, sowing, or site manipulation but with control for disturbances that inhibit natural regeneration
Social remittance: the transmission of new ideas and practices from a migrant back to their household or community of origin
Circular migration: repeated temporary moves by a migrant to a destination with regular returns to their household or community of origin
Remittance: the transmission of money or goods from a migrant back to their household or community of origin
Migrant networks: relationships between migrants and non-migrants that create links between individuals, households, and communities in migrant origins and destinations
Livelihoods framework: the complex relationships among a household’s or individual’s assets, activities, and strategies for gaining a living, in the context of social relations, institutions, and organizations
Initial floristics: a vegetation succession model with synchronous regeneration or release, usually of different species that survive for different lengths of time
Relay floristics: a vegetation succession model with sequential regeneration or release of different species that replace each other over time
Outgrower scheme: a program whereby a forest products company recruits many smallholders to grow wood for it under contract
Payments for ecosystem services (PES): a program, usually governmental, that pays landholders to undertake actions that increase the supply of ecological services from their land
Discrete-choice experiment (DCE): a valuation method that elicits preferences across an intervention’s attributes by asking individuals to choose among differently designed hypothetical interventions
Randomized controlled trial (RCT): an evaluation method that measures an intervention’s impact by randomly assigning it to a subgroup of a random sample of individuals, households, or villages
