# Supplementary material for: Forest Restoration in Low- and Middle-Income Countries
Source: Annu Rev Environ Resour. Author manuscript; Available in PMC 2025 Oct 15. (PMC12520201; doi:10.1146/annurev-environ-012220-020159)
Supplement: Appendix [file NIHMS2048038-supplement-Appendix.pdf]

Supplemental Material: Annu. Rev. Environ. Resour. 2021. 46.
https://doi.org/10.1146/annurev-environ-012220-020159
Forest Restoration in Low- and Middle-Income Countries
Vincent, Curran, and Ashton

Supplemental Appendix: Assessing Medium-Severity Forest Degradation

Jeffrey R. Vincent,[1] Sara R. Curran,[2] and Mark S. Ashton[3]

[1]Nicholas School of the Environment, Duke University, Durham, North Carolina 27708, USA; email: jeff.vincent@duke.edu

[2]Henry M. Jackson School of International Studies, University of Washington, Seattle, Washington 98195, USA; email: scurran@uw.edu

[3]The Forest School at the Yale School of the Environment, Yale University, New Haven, Connecticut 06511, USA; email: mark.ashton@yale.edu

Downloaded from www.annualreviews.org. Guest (guest) IP: 67.168.122.255 On: Sat, 22 Feb 2025 22:25:27

IMPACTS ON NATIVE VEGETATION

The first component of an assessment of medium-severity degradation is to understand the capacity for vegetation to reestablish without planting (1). This component includes understanding where sources of seed exist within the converted landscape and what regeneration and regrowth persists. These factors differ between large-scale conversion for commodity crops and conversion by smallholders. Conversion to commodity crops at large scales can be characterized, in general terms, as leaving no seed source for recolonization and no intact regeneration that can survive and regrow. In such circumstances, residual vegetation is left only as a result of: (*a*) topographies that are inoperable because of steep slopes or rocky or poor soils, or (*b*) environmental laws, if adhered to, which typically protect wetlands and waterways (2). The capacity of the original vegetation to recolonize therefore depends on particular circumstances, as topography, geology and soils, and hydrology vary enormously.

Medium-severity degradation by smallholders occurs at smaller scales and is more incremental and less efficient at removing vegetation. It leaves large amounts of original forest

Downloaded from www.annualreviews.org. Guest (guest) IP: 67.168.122.255 On: Sat, 22 Feb 2025 22:25:27

vegetation within areas that are cultivated or grazed, often purposely because of a forest good (e.g., food, timber) or a forest service (e.g., shade, aesthetics) valued by the landholder. This vegetation adds to vegetation left along bodies of water and wetlands or on inoperable lands. Such converted landscapes have a much greater source of seed for recolonization, more existing regeneration, and more remnant but disturbed forest capable of regrowth (3, 4).

IMPACTS ON SOILS

Impacts on soil structure and fertility form the second component of an assessment of medium-severity degradation. The impacts again vary widely, with clearance and site preparation for large-scale commodity crops generally having a more severe impact on soil structure and fertility due to the use of heavy equipment to clear the land and contour slopes, which disturbs surface organic matter and surface horizons and compacts deeper subsurface soils (5). Surface disturbance is important, especially in wet tropical lowlands where soils retain most of their nutrients in the vegetation or very close to the soil surface and are very susceptible to deep compaction, impeded drainage, and surface erosion because of their already poor structure from millions of years of weathering (6). These soils would be classified as Oxisols in the USDA soil classification system (7).

Soils of wet tropical and subtropical forest regions associated with foothills and mountainous or hilly topography are usually Ultisols (7). Such soils are less weathered, usually younger, better structured, and more fertile (5). They have surface horizons with a sandier soil texture, which in combination with their hilly topography makes them very susceptible to erosion when vegetation is cleared and the soil is cultivated.

Soils associated with more seasonal climates of the tropics and subtropics, where monsoonal, semi-deciduous and deciduous forests originally existed along with woodlands and savannah woodlands, are mostly Alfisols (7). They are more nutrient-rich and structurally more balanced (less erodible) (8). For these reasons, most forests on them have been converted to annual crops and tree crops, in many cases centuries ago (9, 10).

Downloaded from www.annualreviews.org. Guest (guest) IP: 67.168.122.255 On: Sat, 22 Feb 2025 22:25:27

Literature Cited

1. Ashton MS, Kelty MJ. 2018. *The Practice of Silviculture: Applied Forest Ecology*. Hoboken, NJ: Wiley
2. Donald PF. 2004. Biodiversity impacts of some agricultural commodity production systems. *Conserv. Biol.* 18:17–37
3. Holl KD, Loik ME, Lin EH, Samuels IA. 2000. Tropical montane forest restoration in Costa Rica: overcoming barriers to dispersal and establishment. *Restor. Ecol.* 8:339–49
4. Lamb D, Erskine PD, Parrotta JA. 2005. Restoration of degraded tropical forest landscapes. *Science* 310:1628–32
5. Chazdon RL. 2003. Tropical forest recovery: legacies of human impact and natural disturbances. *Perspect. Plant Ecol.* 6:51–71
6. Binkley D, Fisher RF. 2013. *Ecology and Management of Forest Soils*. Oxford: Wiley-Blackwell
7. Brady NC, Weil RR, Weil, RR. 2008. *The Nature and Properties of Soils, Vol. 13*. Upper Saddle River, NJ: Prentice Hall
8. Townsend AR, Asner GP, Cleveland CC. 2008. The biogeochemical heterogeneity of tropical forests. *Trends Ecol. Evol.* 23:424–31

9. DeFries RS, Rudel T, Uriarte M, Hansen M. 2010. Deforestation driven by urban population growth and agricultural trade in the twenty-first century. *Nat. Geosci.* 3:178–81

10. Gibbs HK, Ruesch AS, Achard F, Clayton MK, Holmgren P, et al. 2010. Tropical forests were the primary sources of new agricultural land in the 1980s and 1990s. *P. Natl. Acad. Sci. USA* 107:16732–37

Downloaded from www.annualreviews.org. Guest (guest) IP: 67.168.122.255 On: Sat, 22 Feb 2025 22:25:27
